# Effect of Nonsurgical Mechanical Debridement With or Without Chlorhexidine Formulations in the Treatment of Peri‐Implant Mucositis. A Randomized Placebo‐Controlled Clinical Trial

**DOI:** 10.1111/clr.14405

**Published:** 2025-01-25

**Authors:** Gaetano Isola, Alessandro Polizzi, Maria Santagati, Angela Alibrandi, Vincenzo Iorio‐Siciliano, Luca Ramaglia

**Affiliations:** ^1^ Unit of Periodontology, Department of General Surgery and Medical‐Surgical Specialities University of Catania Catania Italy; ^2^ Unit of Microbiology, Department of Biomedical and Biotechnological Sciences University of Catania Catania Italy; ^3^ Unit of Statistics, Department of Economics University of Messina Messina Italy; ^4^ Unit of Periodontology, Department of Neuroscience, Reproductive Science and Oral Science University of Naples Federico II Naples Italy

**Keywords:** biofilm, bleeding on probing, clinical trial, inflammation, mucositis, smoking

## Abstract

**Objectives:**

To evaluate the treatment of peri‐implant mucositis (PM) using a nonsurgical submarginal peri‐implant instrumentation (NSPI) with or without chlorhexidine (CHX) solutions.

**Methods:**

Fifty‐six patients (28 per group) were randomly assigned to the test (NSPI + 0.12% mouthwash and subgingival CHX irrigation plus tongue brushing with 1% CHX gel) or the control group (NSPI + placebo mouthwash and subgingival placebo irrigation plus tongue brushing with placebo gel). At baseline, 1, 3, 6 months, bleeding on probing (BOP), probing pocket depth (PPD), modified gingival index (mGI), modified plaque index (mPlI), full‐mouth plaque score (FMPS), full‐mouth bleeding score (FMBS), and the proportions of *Aggregatibacter actinomycetemcomitans* , *Porphyromonas gingivalis* , *Tannerella forsythia*, and *Treponema denticola* were recorded. The BOP reduction was set as a primary outcome. Data were analyzed to assess BOP reduction at a 6‐month follow‐up and to identify significant predictors of implant‐site BOP through mixed generalized linear regression.

**Results:**

After 6 months in both groups, a significant reduction of BOP, PD, mPlI, mGI, FMBS, and FMPS was noted (*p* < 0.05). However, at 6 months, the test group was more effective than the controls in reducing median BOP (∆values control/test: 39.3% [95% CI 37.4–42.3] vs. 48.7 [95% CI 46.5–51.2], *p* = 0.044), as well as mPlI (*p* = 0.041) and the proportion of *Treponema denticola* (*p* = 0.039). Moreover, the implant‐sites BOP reduction was significantly influenced by test treatment (*p* < 0.001), history of periodontitis (*p* = 0.003), and a high number of cigarettes/day (*p* = 0.002), the proportion of *Porphyromonas gingivalis* (*p* = 0.021) and *Tannerella forsythia* (*p* = 0.032).

**Conclusions:**

NSPI + CHX showed better results compared to placebo in implant‐sites BOP reduction. The high number of cigarettes/day and the proportion of *Porphyromonas gingivalis* and *T. forsythia* negatively influenced the BOP reduction in PM‐treated patients.

## 1 Introduction

Peri‐implant mucositis (PM) is an inflammatory lesion in the soft tissue around dental implants without peri‐implant bone loss caused by biofilm accumulation in peri‐implant sulcus (Berglundh et al. 2018; Heitz‐Mayfield and Salvi 2018; Renvert et al. 2018), defined as the presence of bleeding (more than one spot at a location around the implant) and/or suppuration on gentle probing, in the absence of bone loss (Tonetti et al. 2023). Although dental implants have been proven to have quite consistent survival rates in a range of 95.7% at 5 years and 92.8% up to 10 years (Gurgel et al. 2017), it is recognized that the increasing marginal bone loss and peri‐implantitis risk remain a substantial possible consequence (Albrektsson et al. 2022). Hence, a recent international consensus report stated that the primary goal of PM treatment is to reduce the bleeding on probing (BOP) around implants (≤ 1 spot/implant is acceptable) (Tonetti et al. 2023). In agreement, the European Federation of Periodontology (EFP) Workshop on the peri‐implant therapy guidelines of 2023 suggested that the treatment of PM should be based on professional nonsurgical sub‐marginal peri‐implant instrumentation (NSPI), together with tailored self‐performed oral hygiene procedures (Carra et al. 2023). To improve the efficacy of NSPI, several authors proposed different professional and patient‐administered local decontaminating chemical measures (e.g., air‐powder abrasives, antibiotics, homecare strategies) with uncertain results concerning NSPI (Iorio‐Siciliano et al. 2023; Salvi and Ramseier 2015). Conversely, positive effects have been proven by conventional PM treatment through a concomitant self‐application of chlorhexidine (CHX) (Ramanauskaite, Fretwurst, and Schwarz 2021).

In addition to mechanical plaque removal, scientific evidence suggested that among lifestyle factors, smoking is considered a dominant negative impact on PM development (Heitz‐Mayfield and Salvi 2018), with a reported frequency of PM ranging from 44.9% to 63.4% (Atieh et al. 2013). Smoking is involved in the pathogenesis of PM through several mechanisms, such as the suppression of the host response on gingival tissues (Ryder, Couch, and Chaffee 2018) and also negatively influences the composition of the peri‐implant microbiota such as *Porphyromonas gingivalis* (Pimentel et al. 2018) and to impact the overall PM diagnosis by masking peri‐implant bleeding and mucosal redness, thus complicating PM diagnosis and treatment (da Silva et al. 2022).

The primary objective of the present study is to compare the efficacy of the NSPI and CHX protocols with respect to NSPI and placebo by means of BOP reduction in the treatment of PM at a 6‐month follow‐up. Furthermore, the influence of possible clinical predictors on the BOP changes was also observed as an exploratory analysis.

## 2 Materials and Methods

### 2.1 Study Design

The study was designed as a randomized, double‐blinded, placebo‐controlled clinical trial with a 6‐month follow‐up. Patients diagnosed with PM (Berglundh et al. 2018; Tonetti et al. 2023) were invited to participate in the study and were randomly allocated to the test (NSPI + protocol with 0.12% CHX mouthwash, subgingival CHX irrigation, and tongue brushing) or the control group (NSPI + placebo mouthwash, subgingival placebo irrigation and tongue brushing). The null hypothesis of no statistical differences in BOP reduction between NSPI approaches was tested, and smoking did not impact implant‐site BOP.

The trial was performed between March 2023 and February 2024. The study was registered on ClinicalTrials.gov registry (ID: NCT05711576) and was conducted in observance of the Principles of the Declaration of Helsinki on experimentation involving human subjects. Written consent was obtained from all patients before the study, and the research protocol was reviewed and approved by the Institutional Review Board of the University of Catania (approval number: 150/2679/2022/PO). The study followed the CONSORT statement guidelines (Appendix S1).

### 2.2 Sample Size Calculation

The sample size calculation was determined using statistical software (G* Power 3.1.9.4). The sample size was calculated using BOP reduction as a primary outcome variable (Iorio‐Siciliano et al. 2023) and considering two groups, a power level of 80% an effect size of 0.80, and a common standard deviation (SD) equal to 3.55 (SD control group equal to 4; SD test group equal to 3.1), a mean reduction of 23.4% in the control group and 20.5% in the test group and a two‐sided level of 5%. Hence, it was fixed a priori that at least 26 subjects per group were needed to achieve a power level of 80%. To avoid potential dropouts, at least 28 subjects per group were enrolled.

### 2.3 Study Participants

All subjects were recruited from the Department of Periodontology, University of Catania, Catania, Italy. Patients with a diagnosis of PM (Tonetti et al. 2023) were invited to participate in the study. The diagnosis of PM was made, in agreement with the last Implant Dentistry Core Outcome Set and Measurement (ID‐COSM) international consensus report (Tonetti et al. 2023), as the presence of bleeding (more than one spot at a location around the implant or presence of a line of bleeding or profuse bleeding at any location) and/or suppuration on gentle probing in the absence of bone loss beyond crestal bone‐level changes resulting from initial bone remodeling.

The inclusion criteria were (1) age ≥ 18 years old, (2) patients with implants with smooth necks supporting cemented or screw‐retained single‐unit crowns with at least one BOP‐positive site, (3) patients with implants placed in both maxilla and mandible, (4) patients with gingivitis or treated periodontitis with the absence of residual PD ≥ 5 mm, and (5) patients with at least of 2 mm of keratinized mucosa (KT) at implant sites.

The exclusion criteria were (1) presence of systemic diseases, (2) pregnancy or lactating, (3) use of inflammatory drugs or antibiotics within 3 months prior to study recruitment, (4) interproximal open contacts between implant restoration and adjacent teeth, and (5) peri‐implantitis (Berglundh et al. 2018). Patients with PM on single and multiple implants were enrolled in the study.

### 2.4 Outcomes Measures

All peri‐implant parameters were assessed at six sites (mesio‐buccal, buccal, disto‐buccal, disto‐oral, oral, and mesio‐oral) using a manual periodontal probe (UNC 15, Hu Friedy, Italy) with a probing force of nearly 0.2 N by a calibrated examiner. The primary outcome variable was the BOP reduction. The number of implants presenting with complete absence of BOP or implants with limited extent of BOP (≤ 1 spot/implant—the presence of a single spot) (Tonetti et al. 2023) was considered treatment success. The secondary outcomes were (1) full‐mouth plaque score (FMPS) (O'Leary, Drake, and Naylor 1972) and full‐mouth bleeding score (FMBS) (Claffey et al. 1990), representing the percentage of sites covered with plaque and with BOP in the entire dentition; (2) presence of plaque at implant sites according to modified plaque index (mPlI) (Mombelli et al. 1987) and to modified gingival index (mGI) (Loe and Silness 1963) recorded at four sites; and (3) pocket probing depth (PPD) measured from the peri‐implant mucosal margin to the bottom of the sulcus.

The smoking patient status was classified as current smokers, ex‐smokers (cessation ≥ 5 years), and never smokers (Isola et al. 2024).

Moreover, the following parameters were also recorded: (1) PM treatment success, considered implants without BOP and (2) number needed to treat (NNT), considered the number of implants needed to be treated to prevent PM.

### 2.5 Randomization, Calibration, and Masking

Patients were randomly assigned to test or control groups using sealed and numbered envelopes. They were randomly assigned to test or control procedures by simple randomization without restrictions and using a 1:1 allocation ratio. Allocation concealment was made using opaque‐sealed envelopes associating even numbers to the test group and odd numbers to the control group. The envelopes were prepared by a clinician not involved in the study using a computerized random number generator (Random.org; www.random.org).

Two examiners (A.P. and V.I.S.) attended a training and calibration session on a total of 10 patients not involved in the trial. Each examiner assessed repeated measurements for BOP and PD once. The agreement between examiners was set using BOP alone. The calibrated examiners were unaware with respect to the test or control procedure.

Probing consistency was considered sufficient if the percentage of agreement within ±5% for BOP between repeated measurements was at least 95%; in this case, the agreement was 94.2%. For the intraexaminer reliability, the kappa coefficients were calculated, reporting high reliability for the first (*k* = 0.84) and the second (*k* = 0.86) examiner. The intraclass correlation coefficient (ICC = 0.829) was calculated for the interexaminer reliability. Both intra‐ and interexaminer reliability were assessed using BOP as the outcome variable. The calibrated examiners were unaware with respect to the test or control procedure.

### 2.6 Clinical Procedures

At baseline, all subjects were properly instructed to achieve adequate self‐performed oral hygiene. A Rinn X‐ray was taken for all implants to exclude implants with peri‐implantitis. In both groups, a full‐mouth supragingival debridement was performed using an ultrasonic device with metal tips, and in the same session, the implants diagnosed with PM were treated by means of NSPI using a sonic scaler with a plastic tip at maximum power (EMS dental, Milan, Italy). Finally, polishing was done using a rotating instrument, and a rubber cup with low abrasive polishing paste was made. Immediately following NSPI, patients were randomized to receive a test or placebo.

Patients of the test group were treated by a clinician following the test protocol, based on brushing the dorsum of the tongue for 1 min using CHX 1% gel (Curasept, Saronno, Italy), rinsing using a CHX 0.12% mouthwash (the patient was asked to rinse for 1 min) (Profar, Carpiano, Italy), subgingival irrigation of implant affected by PM three times within 10 min each using a solution with CHX 0.12% (Profar, Carpiano, Italy) (de Melo Menezes et al. 2021). Patients were asked to use a CHX 0.12% mouthwash (Profar, Carpiano, Italy) twice daily for 2 weeks.

Patients of the control group received, after NSPI, the same treatment protocol and for the same duration as the test group, in which, differently from them, the dorsum of the tongue was brushed with a placebo gel and the solution used to rinse and irrigate was a placebo which had the same flavor and color of CHX solution used in the test group. Patients of both groups were instructed to brush the interproximal area between the implant and teeth by means of cylindrical or conical brushes, or in case of difficult access, a floss threader or specialized floss with a built‐in threader was recommended. Clinical examination was performed at baseline and after 1, 3, and 6 months of treatment.

### 2.7 Microbial Analysis

For the microbial analysis, the four deepest pocket sites per all treated PM implants were chosen in each patient. Microbial samples were harvested at the same pocket implant site by a blinded clinician at baseline and 1, 3, and 6 months after treatment. Samples were obtained using two simultaneously inserted sterile paper points (ISO no. 45). The paper points were positioned for 40 s, removed, and immediately transferred to a sterile tube. Microbiological bacterial concentrations were quantified by real‐time PCR (Applied Biosystems, Foster City, CA, USA), and the detection level was set to 10^3^. The selection of primers was based on previous data sources with information on species‐specific oligonucleotides of the 16S ribosomal RNA using SYBR Green assays (Kirakodu et al. 2008) and standard curves were generated for all the target bacteria using DNA from pure cultures and species‐specific DNA primers (Table S1). Bacterial DNA levels were calculated from the standard curve equations created by tenfold serial dilution of DNA standards. In each patient, all samples were individually analyzed for detection of the count of microbial species proportions such as *A. actinomycetemcomitans* , *P. gingivalis* , *Tannerella forsythia* ( *T. forsythia* ), and *Treponema denticola* ( *T. denticola* ).

### 2.8 Statistical Analysis

Quantitative outcomes were expressed as mean ± standard deviation (SD), standard error, and median and interquartile ranges (Q1–Q3) and categorical variables as absolute frequencies and percentages. A nonparametric approach was applied because the distribution of quantitative outcomes was not expected, such as verified by the Shapiro–Wilk test. In order to perform the intergroup comparison (test vs. control), the Mann–Whitney test was applied for quantitative outcomes and the chi‐squared test for categorical variables. For each quantitative outcome, a comparison among time points (baseline, 1, 3, and 6 months) was performed by using the Fridman test for both groups separately (intragroup analysis), followed by a two‐by‐two comparison performed using the Tukey post hoc test. For the analyses, the patient was set as the test unit. In the case of multiple implants per patient, the median values (95% CI) of all implants with PM were evaluated. The primary outcome was the BOP reduction, whereas the secondary outcome assessed the reduction of PPD, FMPS, FMBS, mPlI, mGI, and the proportion of *A. actinomycetemcomitans* , *P. gingivalis* , *T. forsythia*, and *T. denticola* . Moreover, as an exploratory analysis, it was evaluated the influence of possible clinical predictors of the BOP changes.

Finally, a mixed generalized linear regression model was estimated in order to evaluate significant predictors of implant‐site BOP at all follow‐up sessions (baseline, 1, 3, and 6 months). The fixed effects were represented by the following covariates such as age, gender (male/female), treatment (control group treatment as a reference), smoking, number of cigarettes/day, gingival biotype (thin/thick), history of periodontitis (yes/no), patient compliance, implant location in the maxillary (anterior/posterior), implant location in the mandible (anterior/posterior), suprastructures (cemented/screw‐retained), implant type (bone level/tissue level), implant surface (non modified/modified), PPD, mPlI, mGI, KT, FMBS, FMPS, and the bacterial load of *A. actinomycetemcomitans* , *P. gingivalis* , *T. forsythia*, and *T. denticola* , whereas random effects were represented by multiple implant sites per number of implants per patient. First, uni‐level models were estimated in which a single predictor was tested against the primary outcome (implant‐site BOP at all follow‐up sessions); furthermore, a multi‐level model was estimated by inserting only the variables that were found to be statistically significant in the uni‐level models. Specifically, the potential predictor variables were tested together. Both uni‐ and multi‐level model results were reported as estimation coefficient, 95% Confidence Interval (C.I.), and *p* value.

Statistical analyses were performed using SPSS for Windows package, version 26. A *p* value of < 0.05 was considered statistically significant.

## 3 Results

### 3.1 Sample Characteristics

**FIGURE 1:**
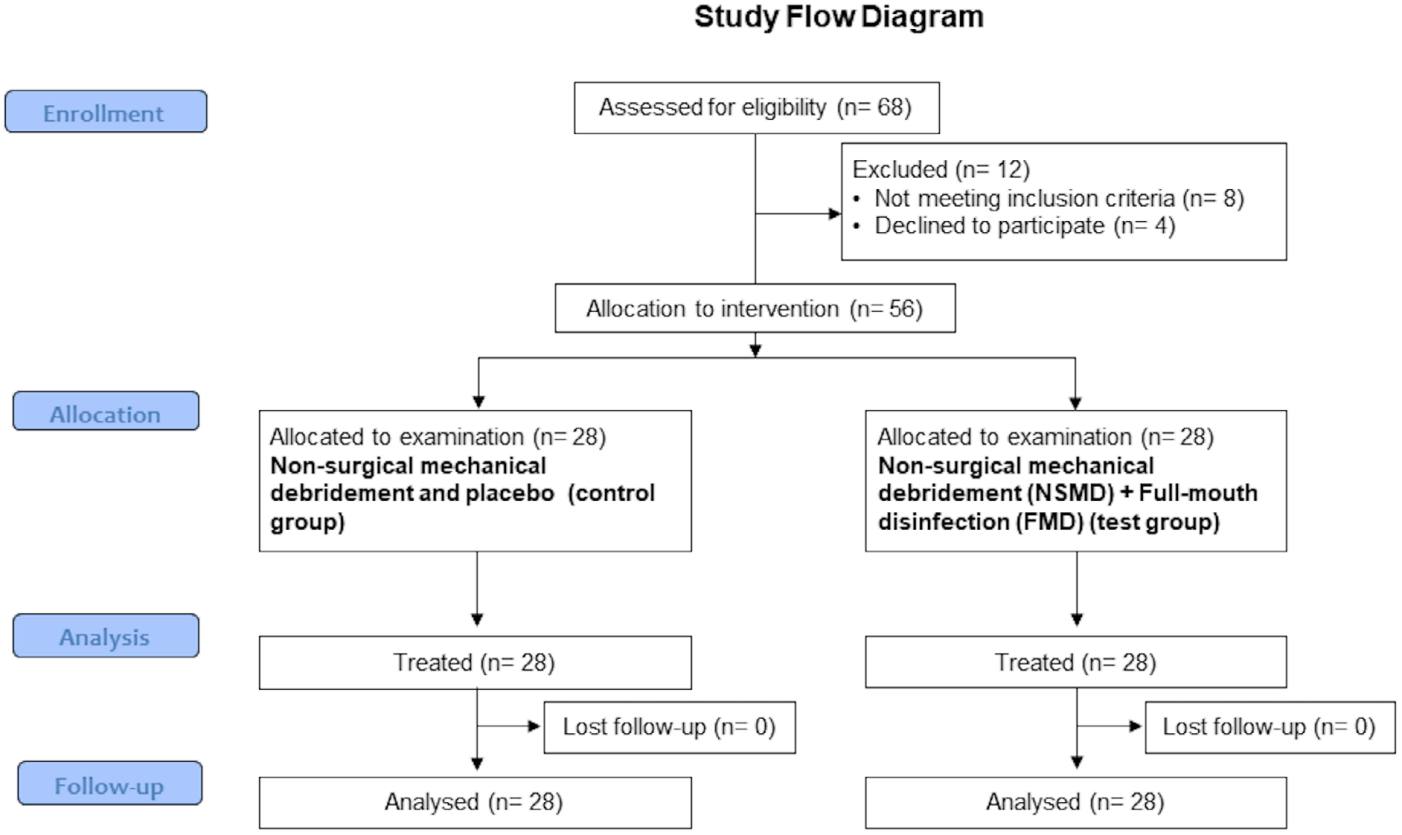
Study flowchart.

For the present study, 68 patients with PM were first enrolled. After the screening, 12 patients were excluded because they did not meet the inclusion criteria (*n* = 8) or refused participation in the study (*n* = 4; Figure 1). A final number of 56 patients (28 patients per group) with 85 implants (40 implants, test group; 35 implants, control group) were enrolled in the present study and included in the analysis (Table 1). During the follow‐up, no implants were lost, and no complications were noted.

**TABLE 1:** Clinical characteristics of the sample divided into control and test groups. Comparison was performed using Mann–Whitney test for quantitative outcomes, and the chi‐squared test for categorical variables.

Parameter	Control group (*n* = 28)	Test group (*n* = 28)	*p*
Gender (male), no. (%)	16 (57.1)	15 (53.4)	0.554
Age, median (IQR)	53.2 (48.6;55.4)	54.5 (51.2–57.8)	0.317
History of periodontitis, no. (%)	6 (21.4)	8 (28.6)	0.224
*Smoking*			
Never smokers, no. (%)	15 (53.6)	13 (46.4)	0.669
Ex‐smokers, no. (%)	3 (10.7)	4 (14.3)	0.104
Current smokers, no. (%)	10 (35.7)	9 (32.1)	0.885
No. cigarettes/day, median (IQR), mean ± SD, standard error	10.2 (8.4–11.6), 10.1 ± 2.1, 0.39	11.3 (8.6–12.4), 10.9 ± 2.3, 0.43	0.296
*Compliance*			
Regular, no. (%)	10 (35.7)	9 (32.1)	0.247
Erratic, no. (%)	14 (50)	14 (50)	0.496
Noncompliers, no. (%)	4 (14.3)	5 (17.9)	0.336
*Gingival biotype*			
Thin, no. (%)	18 (64.3)	15 (53.4)	0.065
Thick, no. (%)	10 (35.7)	13 (46.6)	0.087
KT, mm, median (IQR), mean ± SD, standard error	2.36 (2.12–2.56), 2.19 ± 0.6, 0.11	2.41 (2.15–2.61), 2.44 ± 0.3, 0.05	0.256
Implant location, total no.	40	35	0.074
Anterior maxilla, no. (%)	6 (15)	4 (11.4)	0.105
Posterior maxilla, no. (%)	13 (32.5)	11 (31.4)	0.228
Anterior mandible, no. (%)	5 (12.5)	6 (17.1)	0.471
Posterior mandible, no. (%)	16 (40)	14 (40.1)	0.651
*Implant brand*			
Straumann group, no. (%)	11 (27.5)	7 (20)	0.052
Astra Tech group, no. (%)	16 (40)	13 (37.1)	0.066
BioHorizon, no. (%)	10 (25)	12 (34.3)	0.254
Dentsply Sirona, no. (%)	3 (7.5)	3 (8.6)	0.332
*Treated implant/Patient*			
One implant, no. (%)	20 (71.4)	19 (67.8)	0.254
Two implants, no. (%)	6 (21.4)	7 (25)	0.336
Three implants, no. (%)	2 (7.2)	2 (7.2)	0.995
*Implant surface*			
Modified, no. (%)^a^	26 (92.8)	25 (89.3)	0.587
Nonmodified, no. (%)^b^	2 (7.2)	3 (10.7)	0.664
*Suprastructures*			
Cemented, no. (%)	16 (40)	13 (37.1)	0.154
Screw‐retained, no. (%)	24 (60)	19 (62.9)	0.087
*Implant type*			
Bone level, no. (%)	26 (65)	24 (68.6)	0.449
Tissue level, no. (%)	14 (35)	11 (31.4)	0.227

^a^ Modified surface, TiUnite (Nobel Biocare AB); TiOblast (Dentsply Sirona, Mölndal, Sweden); OsseoSpeed (Dentsply Sirona); SLA (Straumann Institute, Basel, Switzerland); Osseotite (Biomet 3i, Palm Beach, FL, USA); K‐LEAN (Keystone Dental [Lifecore Biomedical]); SBM (ImplantDirect Sybron, California, USA).

^b^ Nonmodified surface, Turned (Nobel Biocare AB, Göteborg, Sweden).

### 3.2 Clinical and Microbiological Outcomes

At baseline, there were no differences among groups for all analyzed parameters (Table 1). Compared to baseline, at 6 months after treatment, both groups reported a significant reduction of median BOP, PPD, FMPS, FMBS, mPlI, mGI, and in the proportion of *P. gingivalis* and *T. forsythia* (*p* < 0.05; Table 2). A further comparison among groups evidenced that, at a 6‐month follow‐up, the test group was more effective than the control group in reducing BOP (test, median: 19.1% [15.4–24.2 95% CI], mean 18.6 ± 4.3%; control, median: 25.3% [21.5–28.6 95% CI], mean 24.9 ± 3.3% *p* = 0.044), mPlI (test, median: 0.64 [0.4–0.7 95% CI], mean 0.7 ± 0.3; control, median, 0.91 [0.5–1.2 95% CI], mean 0.8 ± 0.3, *p* = 0.041), and the count of *T. denticola* (test, median: 2.11 [1.8–2.3 95% CI], mean 2 ± 0.4; control, median: 2.49 [1.9–2.6 95% CI], mean 2.5 ± 0.5, *p* = 0.039), whereas the other parameters were not significantly different (Table 2).

**TABLE 2:** Clinical values of peri‐implant parameters at baseline and at each follow‐up session in both groups.

Parameter	Control group (*n* = 28)			Test group (*n* = 28)		
	Median (95% CI)	Mean ± SD	Standard error	Median (95% CI)	Mean ± SD	Standard error
**BOP**						
Baseline	64.6 (61.4–69.5)	65.1 ± 4.9	0.92	67.8 (60.5–71.2)	66.4 ± 4.5	0.85
1 month	49.2 (45.6–51.4)	48.9 ± 4.6	0.87	53.8 (47.6–58.4)	52.2 ± 4.7	0.88
3 months	30.1 (27.5–31.2)	30.4 ± 2.9	0.54	38.3 (31.2–43.2)	37.7 ± 5.4	1.02
6 months	25.3 (21.5–28.6)	24.9 ± 3.3	0.62	19.1 (15.4–24.2)	18.6 ± 4.3	0.81
Δ 0–6 months	39.3 (37.4–42.3)	40.2 ± 3.5	0.66	48.7 (46.5–51.2)	47.8 ± 4.4	0.83
Intragroup *p* value	0.024			0.039		
**PPD**						
Baseline	3.5 (2.9–4.1)	3.3 ± 0.6	0.11	3.7 (3.2–4.1)	3.6 ± 0.5	0.09
1 month	3.7 (3.2–4)	3.8 ± 0.5	0.09	3.4 (3.1–3.8)	3.3 ± 0.6	0.11
3 months	3.3 (3–3.6)	3.4 ± 0.4	0.07	3.2 (2.9–3.5)	3.1 ± 0.5	0.09
6 months	3.4 (2.9–3.8)	3.2 ± 0.5	0.09	3.6 (3.1–4)	3.5 ± 0.4	0.07
Δ 0–6 months	0.1 (0–0.2)	0.1 ± 0.3	0.05	0.1 (0–0.2)	0.1 ± 0.2	0.03
Intragroup *p* value	0.011			0.047		
**FMPS**						
Baseline	44.5 (41.6–48.9)	44.9 ± 3.2	0.63	45.9 (39.6–49.5)	44.5 ± 4.6	0.87
1 month	24.2 (19.6–27.8)	23.9 ± 3.9	0.73	27.6 (22.5–31.4)	26.5 ± 4.1	0.77
3 months	25.5 (18.5–28.4)	24.9 ± 3.5	0.66	23.1 (19.5–32.5)	22.9 ± 3.9	0.73
6 months	21.9 (17.9–24.5)	22.2 ± 3.8	0.71	19.5 (17.4–22.3)	18.7 ± 3.6	0.68
Δ 0–6 months	22.6 (19.4–23.5)	22.7 ± 3.9	0.73	26.4 (22.4–29.5)	25.8 ± 3.8	0.71
Intragroup *p* value	0.007			0.026		
**FMBS**						
Baseline	33.9 (30.5–36.5)	33.4 ± 5.2	0.98	35.2 (31.2–38.5)	34.4 ± 5.3	1,01
1 month	23.1 (19.5–25.6)	22.9 ± 3.4	0.61	22.3 (20.4–25.3)	21.8 ± 4.1	0.77
3 months	24.5 (21.5–27.8)	23.9 ± 4.2	0.79	25.1 (20.1–28.5)	24.1 ± 3.9	0.73
6 months	24.2 (21.1–26.5)	23.5 ± 4.8	0.90	21.5 (18.6–23.4)	20.9 ± 4.1	0.77
Δ 0–6 months	9.7 (7.7–11.5)	9.9 ± 5.1	0.96	13.7 (10.1–15.2)	13.5 ± 4.6	0.86
Intragroup *p* value	0.048			0.004		
**mPII**						
Baseline	3.35 (2.9–3.6)	3.2 ± 0.3	0.05	3.36 (3.1–3.7)	3.2 ± 0.4	0.07
1 month	2.29 (1.9–2.6)	2.1 ± 0.4	0.07	1.85 (1.6–2.1)	1.7 ± 0.3	0.05
3 months	1.86 (1.6–2)	1.7 ± 0.4	0.07	1.38 (1.1–1.5)	1.4 ± 0.3	0.05
6 months	0.91 (0.5–1.2)	0.8 ± 0.3	0.05	0.64 (0.4–0.7)	0.7 ± 0.3	0.05
Δ 0–6 months	2.44 (1.8–2.9)	2.4 ± 0.2	0.45	2.72 (2.6–3.3)	2.5 ± 0.2	0.03
Intragroup *p* value	0.003			0.014		
**mGI**						
Baseline	3.25 (3–3.4)	3.2 ± 0.3	0.05	3.42 (3.1–3.7)	3.3 ± 0.5	0.09
1 month	2.56 (2.1–2.7)	2.4 ± 0.4	0.07	2.06 (1.8–2.4)	2.1 ± 0.3	0.05
3 months	2.31 (2.2–2.6)	2.2 ± 0.3	0.05	2.12 (1.9–2.3)	2.1 ± 0.4	0.07
6 months	1.42 (1.2–1.6)	1.3 ± 0.2	0.03	1.28 (0.9–1.3)	1.3 ± 0.3	0.05
Δ 0–6 months	1.83 (1.3–2.1)	1.9 ± 0.3	0.05	2.14 (1.5–2.4)	2 ± 0.3	0.05
Intragroup *p* value	0.026			0.007		
*A. actynomycencomitans*						
Baseline	0.53 (0.4–0.6)	0.4 ± 0.2	0.03	0.61 (0.4–0.7)	0.5 ± 0.2	0.03
1 month	0.41 (0.2–0.6)	0.4 ± 0.2	0.03	0.39 (0.2–0.5)	0.4 ± 0.3	0.05
3 months	0.39 (0.2–0.6)	0.4 ± 0.3	0.05	0.47 (0.4–0.6)	0.5 ± 0.2	0.03
6 months	0.45 (0.2–0.5)	0.5 ± 0.2	0.03	0.39 (0.3–0.5)	0.4 ± 0.2	0.03
Δ 0–6 months	0.08 (0–0.1)	0.1 ± 0.2	0.03	0.22 (0.1–0.3)	0.3 ± 0.2	0.03
Intragroup *p* value	0.254			0.069		
*P. gingivalis*						
Baseline	3.23 (2.9–3.5)	3.1 ± 0.3	0.05	2.84 (2.6–2.9)	2.7 ± 0.3	0.05
1 month	3.02 (2.8–3.3)	3.1 ± 0.2	0.03	2.55 (2.3–2.7)	2.6 ± 0.3	0.05
3 months	2.53 (2.2–2.7)	2.4 ± 0.2	0.03	3.12 (2.9–3.5)	3.1 ± 0.3	0.05
6 months	2.09 (1.8–2.1)	2.1 ± 0.2	0.03	2.25 (2.3–2.6)	2.3 ± 0.3	0.05
Δ 0–6 months	1.14 (0.9–1.3)	0.8 ± 0.2	0.03	0.59 (0.3–0.6)	0.4 ± 0.2	0.03
Intragroup *p* value	0.021			0.048		
*T. forsythia*						
Baseline	3.87 (3.5–4.1)	3.7 ± 0.3	0.05	3.75 (3.5–4)	3.7 ± 0.4	0.07
1 month	3.31 (2.9–3.6)	3.2 ± 0.3	0.05	3.24 (2.8–3.4)	3.3 ± 0.3	0.05
3 months	3.15 (3–3.4)	3.1 ± 0.2	0.03	3.09 (2.8–3.2)	3.1 ± 0.3	0.05
6 months	2.61 (2.3–2.9)	2.5 ± 0.2	0.03	2.55 (2–2.6)	2.6 ± 0.3	0.05
Δ 0–6 months	1.26 (0.9–1.4)	1.2 ± 0.4	0.07	1.2 (0.9–1.4)	1.1 ± 0.2	0.03
Intragroup *p* value	0.002			0.015		
*T. denticola*						
Baseline	3.11 (2.9–3.3)	3.2 ± 0.3	0.05	2.85 (2.6–2.9)	2.9 ± 0.3	0.05
1 month	2.82 (2.6–3.2)	2.9 ± 0.4	0.07	2.28 (1.9–2.3)	2.3 ± 0.4	0.07
3 months	2.56 (2.3–2.8)	2.6 ± 0.3	0.05	2.47 (2.3–2.7)	2.5 ± 0.3	0.05
6 months	2.49 (1.9–2.6)	2.5 ± 0.5	0.09	2.11 (1.8–2.3)	2 ± 0.4	0.07
Δ 0–6 months	0.62 (0.4–0.8)	0.7 ± 0.3	0.05	0.74 (0.5–0.8)	0.9 ± 0.3	0.05
Intragroup *p* value	0.225			0.068		

*Note*: Results are presented such as median (95% CI), mean and standard deviation (SD), and standard error. For bacteria results, the level of detection was set to 10^3^ bacteria. The bacteria levels were detected in all PM analyzed sites. Comparison was performed using the Fridman test (intragroup analysis) and the Tukey post hoc test (two‐by‐two comparison).

At 1 month (*p* = 0.047) and at 6 months (*p* = 0.037), in comparison with the control group, in the test group, there was a significantly higher number of implants, which reached a maximum of 1 BOP‐positive site (Table 3). Moreover, the mean NTT was 15.2 at 1 month, 22.3 at 3 months, and 11.6 at 6 months (Table 4). Moreover, Δ values between baseline and 6 months after treatment in both groups evidenced that the test group experienced a significant reduction of BOP (*p* = 0.029), FMBS (*p* = 0.042), mPII (*p* = 0.045), *A. actinomycetemcomitans* (*p* = 0.012), and *P. gingivalis* (*p* = 0.006).

**TABLE 3:** Number and percentage of patients with BOP‐negative implants (treatment success) at 1‐, 3‐, and 6‐month follow‐ups.

Follow‐up session	Control group (*n* = 35 implants)	Test group (*n* = 40 implants)	*p*	NNT
**BOP‐negative implants**				
Baseline, no. (%)	29 (82.3)	33 (82.5)	0.374	
1 month, no. (%)	22 (62.9)	16 (40)	0.047	22.3
3 months, no. (%)	17 (48.6)	15 (37.5)	0.247	12.4
6 months, no. (%)	15 (42.9)	10 (25)	0.037	11.6
Intragroup *p* value	0.041	0.009		

*Note*: Treatment success was defined as patients with implants without BOP (≤ 1 spot/implant). Comparison performed using chi‐squared test.

Abbreviation: NNT, number needed to treat.

**TABLE 4:** Uni‐ and multi‐level mixed generalized linear regression model for implant‐sites BOP at all follow‐up sessions.

Parameter	Uni‐level model			Multi‐level model		
	β	95% CI	*p*	β	95% CI	*p*
**Fixed effects**						
Treatment	−3.48	−5.250;−1.709	< 0.001	−3.27	−4.245; −1.954	< 0.001
Treatment × time	−4.98	−5.236;−3.254	0.057	—	—	—
Age	−2.48	−3.456;−1.236	0.103	—	—	—
Gender	0.96	−0.286;1.884	0.055	—	—	—
Smoking	3.67	−4.346;1.679	0.009	3.55	2.874;3.745	0.022
Cigarettes/day	5.44	4.984; 5.805	0.002	4.42	3.125;4.587	0.036
Gingival biotype	−0.84	−1.223;0.782	0.043	−0.55	−0.956;‐0.214	0.336
History of periodontitis	−4.41	−5.986;1.678	0.003	−3.25	−2.845;1.421	0.019
Patient compliance	−5.23	−5.556;‐2.792	0.059	—	—	—
Implant location in maxilla	1.22	0.983;2.278	0.236	—	—	—
Suprastructures	−2.45	−3.443;2.209	0.066	—	—	—
Implant type	1.89	1.113;2.346	0.079	—	—	—
Implant surface	−2.03	1.557;2.974	0.135	—	—	—
PPD	4.55	2.119;6.024	0.032	4.21	2.356;5.247	0.036
mPlI	1.16	−1.385;1.985	0.023	1.04	0.556;1.332	0.417
mGI	2.06	0.441;1.657	0.053	—	—	—
KT	−1.04	−2.345;‐0.546	0.066	—	—	—
FMBS	2.45	1.034;3.456	0.041	2.21	1.524;3.114	0.043
FMPS	3.32	2.206;4.258	0.039	2.87	1.965;4.125	0.041
*A. actynomycencomitans*	3.45	2.568;4.257	0.118	—	—	—
*P. gingivalis*	0.95	−1.344;1.783	0.021	0.88	−0.956;1.521	0.029
*T. forsythia*	3.45	2.336;4.456	0.032	3.12	2.115;4.198	0.045
*T. denticola*	1.57	−0.709;3.124	0.245	—	—	—
**Random effects**						
Multiple implant sites per number of implants per patient	0.856	0.046;15.884	0.502	0.745	0.365;8.657	0.346

*Note*: For treatment, control was set as a reference; for gender, female was set as a reference; for gingival biotype, thin type was set as a reference; for the history of periodontitis, no periodontitis was set as a reference; for patient compliance, regular was set as a reference; for implant location in maxilla and mandible, anterior was set as a reference; for suprastructures, cemented was set as a reference; for implant type, bone level was set as a reference, for implant surface, modified was set as a reference. Results were expressed as beta coefficient estimation (β), 95% confidence interval (95% CI), and *p* value.

The mixed generalized linear regression analysis aimed at evaluating possible significant predictors of implant‐site BOP at all follow‐up sessions in both groups of patients highlighted that reduced median BOP values over the follow‐up session at uni‐ and multi‐level estimation models were significantly influenced by the experimental treatment performed in the test group (*p* < 0.001 for both models). More specifically, at the uni‐level models, the analysis evidenced that the BOP reduction was significantly influenced by the following fixed effects such as smoking (*b* = 3.67; *p* = 0.009), number of cigarettes/day (*b* = 5.44; *p* = 0.002), thin gingival biotype (*b* = −0.84; *p* = 0.043), high PPD (*b* = 4.55; *p* = 0.032), high mPII (*b* = 1.16; *p* = 0.023), high FMBS (*b* = 2.45; *p* = 0.041), if the patient had no history of periodontitis (*b* = −4.41; *p* = 0.003), high FMPS (*b* = 3.32; *p* = 0.039), and by high proportions of *P. gingivalis* (*b* = 0.95; *p* = 0.021) and *T. forsythia* (*b* = 3.45; *p* = 0.032). Furthermore, the multi‐level analysis evidenced that the BOP reduction was also significantly influenced by the following fixed effects such as smoking (*b* = 3.55; *p* = 0.022), number of cigarettes/day (*b* = 4.42; *p* = 0.022), high PPD (*b* = 4.21; *p* = 0.036), high FMBS (*b* = 2.21; *p* = 0.043), high FMPS (*b* = 2.87; *p* = 0.041), if the patient had no history of periodontitis (*b* = −3.25; *p* = 0.019), and by high proportions of *P. gingivalis* (*b* = 0.88; *p* = 0.029) and *T. forsythia* (*b* = 3.12; *p* = 0.045; Table 4).

At the same time, the mixed generalized random effects, represented by the multiple implant sites per number of implants per patient, did not influence, in both uni‐ and multi‐level models, the reduction of BOP median values over time in all enrolled patients (uni‐level: *b* = 0.856, *p* = 0.502; multi‐level: *b* = 0.745, *p* = 0.346), as well as the interaction of treatment and time of treatment (uni‐level: *b* = −4.98, *p* < 0.001; multi‐level: *b* = −5.06; *p* < 0.001).

## 4 Discussion

The main objective of the present study was to compare the efficacy of NSPI protocols with or without CHX for the treatment of PM at a 6‐month follow‐up using the BOP reduction, whereas the secondary outcome evaluated the influence of possible predictors on the BOP changes among all follow‐up sessions. In the present study, both analyzed treatments with traditional subgingival instrumentation with or without the use of CHX were efficient in reducing clinical and microbial outcomes in PM patients. However, in both treatment groups, the implants with PM were treated using the same clinical procedure.

In this regard, although different devices were proposed to decontaminate the implant necks without modification of the titanium surface (Cafiero et al. 2017), in this trial, a sonic scaler with a plastic tip was used; the choice was based on previous evidence that reported better outcomes when plastic tips were used for PM treatment (Blasi et al. 2016).

In the present study, the test treatment was more effective in reducing median BOP in all follow‐up sessions, even when counted for the multiple implant sites per number of implants per patient, and was more effective in reducing median mPlI and the median count of *T. denticola* , whereas the other parameters, such as mGI, were not different among groups. These beneficial results for the PM treatment are in agreement with some studies that reported a reduction of PM when patients were treated with NSPI and CHX through a complete PM healing in 43.7% of the treated implants (Maximo et al. 2009), but in contrast with the recent systematic review (Verket et al. 2023) which no reported any greater beneficial effects among PM treatments with a quite well reduction in BOP severity (0.61–0.84) and PM treatment success (12.5%). In agreement with the present results, some evidence has reported CHX to be effective in the reduction of PPD as an adjunct to traditional NSPI (Ramanauskaite, Fretwurst, and Schwarz 2021), with results ranging from −0.04 to 2.40 mm CI (Ye et al. 2023) or in the decrease of mGI, a clinical parameter strictly related to inflammatory status in PM patients in the short term (Alqutub et al. 2023), thus indicating a quite positive impact of CHX on PM. Moreover, a recent systematic review on this topic reported that the synthesis of data revealed no clear superiority regarding the use of adjunctive treatments such as CHX when considering change in BOP and PPD reduction in the long term of PM treatment, which may be due to the heterogeneity in the presence/absence of smokers in the analyzed studies (Dommisch et al. 2023; Gennai et al. 2023). Based on these findings, the present study was designed to further investigate the hypothesis that also smoking levels could influence the clinical and microbiological outcomes of NSPI of PM performed with or without the use of CHX.

More importantly, the present study results evidenced, through regression model, that reduced BOP levels were significantly influenced, in the multi‐level model analysis, by treatment with CHX (test group), smoking status and high PPD, *T. forsythia* levels, and history of periodontitis. In agreement, a cross‐sectional study evidenced a dose‐dependent effect (OR 0.773) of smoking on BOP, even adjusting data for PPD, plaque, socioeconomic status and body mass index, in comparison to plaque‐negative periodontal sites (Singh and Gangwar 2021). Thus, several preliminary evidence report that smoking is capable of determining a direct occurrence of gingival inflammation revealed through gingival bleeding and augmented inflammatory response of peri‐implant tissues. Conversely, some recent studies showed that BOP exhibited less frequently in smokers despite a higher % of PI and PPD, even though these outcomes were not confirmed in a multivariate analysis (Alasqah et al. 2019; ArRejaie et al. 2019). Concerning the effect on PM and peri‐implantitis, it was reported that smoking, together with a history of periodontitis, also impacts peri‐implant inflammation in the long run (Annunziata et al. 2024). In this regard, Mangano et al. (2016), in a retrospective study on 1740 implants placed in 885 patients, found that heavy smoking (defined as 15 cigarettes/day) was associated, in agreement with the present results, on peri‐implant inflammation and an increase of about two times in early implant failure (6.1%).

In this regard, the multi‐level regression model evidenced that a high number of cigarettes/day, together with high PPD, FMBS, FMPS, the patient's history of periodontitis, and high concentrations of *P. gingivalis* and *T. forsythia* , significantly influenced the BOP reduction after treatment at all follow‐up sessions in all enrolled patients. Several evidence have reported that smoking may also induce a negative impact on the peri‐implant microbiome and alter the host–microbial interaction response (Monje, Kan, and Borgnakke 2023). In general, the reduced BOP tendency in smokers has also been shown to may negatively impact both diagnosis of peri‐implant health, periodontitis (Isola et al. 2025) and PM, thus also accelerating the onset of peri‐implantitis (Insua et al. 2023).

The present study has, however, several limitations that should be addressed. One is regarding the number of patients. A more significant number of enrolled patients could have determined a higher power to detect better significant differences between treatment groups. Another limitation was the change in the primary objective during the trial. Initially, the primary objective was to investigate the impact of tobacco smoking on the resolution of peri‐implant mucositis after nonsurgical mechanical debridement with or without CHX. Although some factors (i.e., smoking habit) could negatively affect the healing, the resolution of PM depends on biofilm removal. For these reasons, the main objective was changed during the investigation. Moreover, a longer follow‐up would have been desirable to better evaluate the long‐term impact of the differential results on PM in the analyzed sample.

## 5 Conclusion

Within the limitations, the present study evidenced that both clinical procedures improve clinical and microbiological parameters at a 6‐month follow‐up. However, applying the test protocol to NSPI resulted in a statistically significant reduction of BOP, mPll, and the proportion of *T. denticola* . In addition, history of periodontitis, smoking status, number of cigarettes/day, high PPD, FMBS, FMPS, and high concentrations of *P. gingivalis* and *T. forsythia* negatively influenced the BOP reduction in both groups. However, further studies with a larger sample, and a longer follow‐up are needed to better understand the test protocol's effectiveness in treating PM.

## Author Contributions

**Gaetano Isola:** conceptualization, writing – review and editing, writing – original draft, funding acquisition. **Alessandro Polizzi:** methodology, investigation, formal analysis. **Maria Santagati:** methodology, investigation, formal analysis, data curation, supervision. **Angela Alibrandi:** formal analysis, software, data curation, methodology. **Vincenzo Iorio‐Siciliano:** conceptualization, methodology, writing – original draft. **Luca Ramaglia:** supervision, visualization, writing – review and editing.

## Ethics Statement

This study was approved by the Institutional Review Board of the University of Catania (approval number: 150/2679/2022/PO). All procedures performed in studies involving human participants were in accordance with the ethical standards of the Institutional Review Board and with the 1964 Helsinki Declaration on experimentation involving human subjects and its later amendments or comparable ethical standards.

## Conflicts of Interest

The authors declare no conflicts of interest.

## Supporting information


**Appendix S1.** CONSORT 2010 checklist of information to include when reporting a randomized trial.

**Table S1.** 16S rDNA bacterial primers used in real‐time PCR in this study (Kirakodu et al. 2008).

## Data Availability

The data that support the findings of this study are openly available in clinicaltrials.gov, reference number NCT05711576.
