# Supplementary material for: Effect of Nonsurgical Mechanical Debridement With or Without Chlorhexidine Formulations in the Treatment of Peri‐Implant Mucositis. A Randomized Placebo‐Controlled Clinical Trial
Source: Clin Oral Implants Res. 2025 Jan 25;36(5):566–77. doi: 10.1111/clr.14405 (PMC12066909; doi:10.1111/clr.14405)
Supplement: Supplementary file 2 — Table S1. 16S rDNA bacterial primers used in real‐time PCR in this study (Kirakodu et al. 2008). [file CLR-36-566-s002.docx]

**Supplementary Table 1.** 16S rDNA Bacterial Primers Used in Real Time PCR in this study (Kirakodu, Govindaswami, Novak, Ebersole, & Novak, 2008)

| **Organism** | **Sequence** | **Size (bp)** | **Annealing Temperature** |
| --- | --- | --- | --- |
| *Aggregatibacter actinomycetemcomitans* | CTAGGTATTGCGAAACAATTTG | 262 | 55 |
|  | CCTGAAATTAAGCTGGTAATC |  |  |
| *Porphyromonas gingivalis* | AGGCAGCTTGCCATACTGCG | 404 | 60 |
|  | ACTGTTAGCAACTACCGATGT |  |  |
| *Treponema denticola* | TAATACCGAATGTGCTCATTTACAT | 316 | 59 |
|  | TCAAAGAAGCATTCCCTCTTCTTCTTA |  |  |
| *Tannerella forsythia* | GCGTATGTAACCTGCCCGCA | 641 | 62 |
|  | TGCTTCAGTGTCAGTTATACCT |  |  |
